# Real-world application of ATA Guidelines in over 600 aspirated thyroid nodules: is it time to change the size cut-offs for FNA?

**DOI:** 10.1530/ETJ-22-0163

**Published:** 2022-10-10

**Authors:** Stamatina Ioakim, Akheel A Syed, George Zavros, Michalis Picolos, Luca Persani, Angelos Kyriacou

**Affiliations:** 1 CEDM, Centre of Endocrinology, Diabetes & Metabolism, Limassol, Cyprus; 2 Medical School, University of Milan, Milan, Italy; 3 Department of Diabetes, Endocrinology & Obesity Medicine, Salford Royal NHS Foundation & University Teaching Trust, Salford, UK; 4 Division of Diabetes, Endocrinology & Gastroenterology, Faculty of Biology, Medicine and Health, The University of Manchester, Manchester, UK; 5 Alithias Endocrinology Centre, Nicosia, Cyprus; 6 Department of Medical Biotechnology and Translational Medicine, University of Milan, Milan, Italy; 7 Department of Endocrinology and Metabolic Diseases, IRCCS Istituto Auxologico Italiano, Milan, Italy; 8 Medical School, European University of Cyprus, Nicosia, Cyprus

**Keywords:** thyroid nodules, ultrasound risk stratification, fine-needle aspiration biopsy, size limits, size cut-offs, thyroid malignancy, thyroid cancer, thyroid carcinoma, papillary thyroid carcinoma

## Abstract

**Background:**

The 2015 American Thyroid Association (ATA) Guidelines recommend the following size cut-offs based on sonographic appearances for subjecting nodules to fine-needle aspiration (FNA) biopsy: low risk: 15 mm and intermediate risk and high risk: 10 mm.

**Objective:**

We conducted a ‘real-world’ study evaluating the diagnostic performance of the ATA cut-offs against increased thresholds, in the interest of safely limiting FNAs.

**Methods:**

We performed a retrospective analysis of prospectively collected data on 604 nodules which were sonographically risk-stratified as per the ATA Guidelines and subsequently subjected to ultrasound-guided FNA. Nodules were cytologically stratified into ‘benign’ (Bethesda class 2) and ‘non-benign’ (Bethesda classes 3–6). We obtained the negative predictive value (NPV), accuracy, FNAs that could be spared, missed ‘non-benign’ cytologies and missed carcinomas on histology, according to the ATA cut-offs compared to higher cut-offs.

**Results:**

In low-risk nodules, the high performance of NPV (≈91%) is unaffected by increasing the cut-off to 25 mm, and accuracy improves by 39.4%; 46.8% of FNAs could be spared at the expense of few missed B3–B6 cytologies (7.9%) and no missed carcinomas. In intermediate-risk nodules, a 15 mm cut-off increases the NPV by 11.3% and accuracy by 40.7%. The spared FNAs approach 50%, while B3–B6 cytologies are minimal, with no missed carcinomas. In high-risk nodules, low NPV (<35%) and accuracy (<46%) were obtained regardless of cut-off. Moreover, the spared FNAs achieved at higher cut-offs involved numerous missed ‘non-benign’ cytologies and carcinomas.

**Conclusion:**

It would be clinically safe to increase the ATA cut-offs for FNA in low-risk nodules to 25 mm and in intermediate-risk nodules to 15 mm.

## Introduction

Epidemiological studies suggest that approximately 50–70% of adults in the general public and across different geographic locations harbour thyroid nodules if examined by thyroid ultrasound scan (USS), while 20% have a nodule that exceeds 10 mm (1, 2, 3). Autopsy series revealed that up to 11% of people bear small intrathyroidal carcinomas (4). Despite this, according to guidelines, only approximately 5% of all thyroid nodules will harbour malignant disease (5). Reassuringly, a recent long-term study of a 17,500-patient cohort with nodules greater than 10 mm in size showed an even lower risk of malignancy at 1.1% (6). The latter study differed from previous studies in that there was less selection bias and papillary microcarcinomas were excluded from the rate of malignancy calculation.

Differentiated thyroid carcinoma is by far the most common cause of thyroid cancer and carries an excellent prognosis with 10-year survival rates reaching 99%, albeit with a 5–20% risk of recurrence (5). As such, physicians managing thyroid nodules need to identify the patients with ‘clinically significant’ carcinomas without subjecting too many people to invasive procedures i.e. fine-needle aspiration (FNA) biopsies and thyroidectomies. Thyroid FNA can provide valuable information for the management of thyroid nodules when clearly indicated. Although major complications are infrequent, FNA remains an invasive procedure and should therefore be limited to cases that would benefit from altered clinical management. Indeed, a national screening programme in South Korea did not show any reduction in mortality despite a substantial rise in thyroidectomies and differentiated thyroid carcinoma diagnoses (7).

Diagnostic ultrasound stratification systems function as ‘rule-out tools’ that prevent unnecessary FNAs in thyroid nodules. There is evidence that the application of such systems in routine clinical practice reduces unnecessary FNAs while not unduly increasing the rate of missed cancers or cancer-related morbidity and mortality (8, 9).

Various risk stratification systems (RSSs) exist as proposed by different medical societies (5, 10, 11, 12, 13, 14, 15). Probably the most widely adopted stratification system is the one proposed by the American Thyroid Association (ATA) (10). The ATA recommends the following nodule size cut-offs based on the risk category, as determined by the sonographic appearances: low-risk nodules should be biopsied at 15 mm and intermediate and high-risk nodules at 10 mm. In a prior comparative study of five ultrasound RSSs, ACR-TIRADS outperformed other systems in safely and substantially reducing the number of FNAs carried out (16). The comparative size cut-offs used by ACR-TIRADS for recommending FNA are as follows: low-risk nodules at 25 mm, intermediate-risk nodules at 15 mm and high-risk nodules at 10 mm (12). The corresponding size cut-offs of the EU-TIRADS system of the European Thyroid Association (ETA) are at >20 mm for low-risk nodules, >15 mm for intermediate-risk nodules and >10 mm for high-risk nodules (11). Table 1 shows the recommended size cut-offs and variation between guidelines.

**Table 1:** Comparison of thyroid nodule size cut-offs for FNA according to the sonographic risk stratification systems of various guidelines.

	Low risk (mm)	Intermediate risk (mm)	Highrisk (mm)
ATA	≥15	≥10	≥10
ACR TI-RADS	≥25	≥15	≥10
ETA EU-TIRADS	>20	>15	>10
AACE/ACE/AME	>20	>20	>10
SIE	≥20	≥20	≥10
K-TIRADS	≥15	≥10	≥10
BTA	Avoid FNA^a^	≥10	≥10
Index study	≥25	≥15	≥10

For simplicity and comparability, we divided the thresholds into three risk categories even though the actual terminology used differs between guidelines (e.g. ATA uses low suspicion, intermediate suspicion, high suspicion, ACR-TIRADS use TR3 mildly suspicious, TR4 moderately suspicious, TR5 highly suspicious and BTA uses U2, U3, U4–5 for what we defined as low risk, intermediate risk and high risk, respectively). Nonetheless, the guidelines allow for a degree of flexibility given the presence of factors such as strong family history and patient preference. Furthermore, the general consensus is that FNA is indicated in nodules measuring less than 10 mm provided they have high-risk appearance on USS and associated capsule infiltration or highly suspicious lymphadenopathy.

^a^The BTA Guidelines state that ‘US appearances that are indicative of a benign nodule (U1–U2) should be regarded as reassuring not requiring fine-needle aspiration cytology (FNAC), unless the patient has a statistically high risk of malignancy’.

AACE, American Association of Clinical Endocrinologists; ACE, American College of Endocrinology; ACR, American College of Radiology; AME, Associazione Medici Endocrinologi; ATA, American Thyroid Association; BTA, British Thyroid Association; ETA, European Thyroid Association; K-TIRADS, Korean-TIRADS; SIE, Italian Society of Endocrinology; TIRADS, Thyroid Imaging Reporting and Data System.

It is yet unknown whether the diagnostic performance of the ATA Guidelines can be improved simply by adopting higher cut-offs for recommending FNA without any other alterations to the guidelines.

Our aim was to assess the correlation between sonographic and cytological risk stratification with particular emphasis on the size cut-offs. We sought to investigate whether an increase in the ATA-proposed cut-offs could safely limit the number of FNAs carried out. The alternative hypothesis was that by increasing the size threshold in the ATA Guidelines for aspirating thyroid nodules, this would result in fewer FNAs performed with no significant increase in the amount of missed high-risk cytologies or malignancies on histology.

## Materials and methods

We conducted a retrospective observational study of prospectively collected data where we sonographically stratified all nodules as per the ATA Guidelines prior to performing ultrasound-guided FNA.

### Study characteristics

A real-world study design was implemented whereby all patients underwent follow-up and FNA as per our institutional protocol with no deviation from routine clinical practice and with minimal exclusions. The study participants comprised endocrinology outpatients presenting with signs or symptoms indicative of thyroid disease and evident thyroid nodules on ultrasound examination. Our patient cohort consisted of consecutive patients who underwent FNA (AK) and consecutive patients referred for pre-operative lymph node mapping (GZ, MP). The latter group comprised 10% of the overall cohort and the majority had nodules with Bethesda classification 5 (B5) and B6 cytology. A* post hoc* ‘sensitivity’ analysis was also performed excluding this group of patients that were referred for pre-operative lymph node mapping.

The ultrasound scan (USS) assessment of all nodules was performed by two experienced sonographers (AK and GZ) using the Esaote MyLab Seven (Italy) ultrasound machine system with a 13 MHz linear-array transducer. The nodules were classified in real-time into one of five risk categories based on suspicious sonographic appearances (benign, very low suspicion, low suspicion, intermediate suspicion and high suspicion), as per the ATA Guidelines where higher risk categories are associated with increasing risk of malignancy (10). Aside from size (maximum diameter), each nodule was assessed based on the following morphological characteristics: degree of echogenicity (anechoic/hypoechoic/hyperechoic, mildly/markedly), composition (cystic/solid/mixed/spongiform), margins (well/poorly defined/irregular), presence and type of calcifications (macro/microcalcifications), extrathyroidal extension, capsule integrity, shape based on short-to-long axis ratio and associated lymphadenopathy. Elastography is provided in the Supplementary materials (see section on supplementary materials given at the end of this article). Whenever the sonographic appearances did not clearly fit a specific ATA category, this was addressed by reaching consensus between sonographers (AK and GZ or AK, GZ and MP for patients referred by MP) adopting a ‘best-fit’ principle. For example, isoechoic and poorly defined nodules were classified as low risk, whereas mildly hypoechoic and poorly defined nodules were usually considered intermediate risk, based on the overall sonographic presentation. Rarely, when the consensus was for in-between categories (e.g. low-to-intermediate risk), the higher risk category was attributed for the purposes of the study. Twenty-four nodules in total were described with the following ambiguous appearances: isoechoic and poorly defined, mildly hypoechoic, mildly hypoechoic and well defined, or isoechoic with macrocalcifications. Out of these 24 nodules, 13 and 10 nodules were sonographically characterized as low and intermediate risk, respectively, and all were classified as benign, Bethesda class 2 (B2) on cytology. Only one nodule which had ill-defined clusters of microcalcifications was characterized as high risk and was classified as Bethesda class 6 (B6) on cytology (17).

Ultrasound-guided FNA was subsequently performed to triage nodules for further surgical intervention. A limited number (15%) of nodules measuring less than 10 mm were biopsied due to concerns pertaining to capsule infiltration and/or presence of suspicious lymph nodes and/or family history of thyroid carcinoma and/or patient preference, reflecting real-world practice, as well as the fact that a number of these patients were referred for pre-operative lymph node mapping (Supplementary Table 1).

Free-needle technique was employed using a 23 G needle without aspiration (AK and GZ) or aspiration via a syringe using a 25 G needle (MP). In case of cystic nodules or insufficient material obtained via free-hand technique, aspiration with a syringe was applied by all practitioners. One to three passes per nodule were performed depending on the apparent adequacy of the sample and how well the procedure was tolerated by the patient. None of the patients experienced any major bleeding nor other significant complications consequent to the aspiration.

The reference standard diagnosis we considered was the cytology results, where nodules were classified into ‘benign’ or ‘non-benign’. The benign category consisted of nodules with Bethesda Classification 2 (B2) cytology (benign), while the non-benign category was defined by the presence of B3–B6 cytology results (atypia of undetermined significance or follicular lesion of undetermined significance, follicular neoplasm or suspicious for a follicular neoplasm, suspicious for malignancy and malignant, respectively). Hence, Bethesda class 2 cytologies were considered as ‘benign’ nodules (true negative) and Bethesda class 3, 4, 5 and 6 cytologies were considered as ‘non-benign’ (true positive). All cytology samples were reviewed by experienced consultant cytopathologists and assigned one of six diagnostic categories of the 2017 Bethesda System for Reporting Thyroid Cytopathology (TBSRTC).

We included thyroid nodules fulfilling the following criteria: (i) documented USS classification as per ATA Guidelines, (ii) subsequent cytology, (iii) available size information, (iv) biopsied between 1 January 2016 and 22 July 2022. Nodules with non-diagnostic cytology, Bethesda class 1 (B1), were excluded. The clinical management of patients with ‘Benign’ nodules (B2 cytology) involved annual follow-up for observation. Post-thyroidectomy specimens were reviewed by experienced consultant histopathologists and staged as per the TNM system of the American Joint Committee on Cancer (AJCC) (7th edition until 31 December 2017, 8th edition as from 1 January 2018). We subsequently re-assessed the reports and modified the staging where necessary as per the 8th edition indications (18).

The primary outcome measure of our study was to assess the diagnostic performance of the ATA-proposed cut-offs as compared to higher cut-offs in terms of negative predictive value (NPV), accuracy, FNAs that can be spared and missed high-risk cytologies or malignancies on histology. Emphasis was placed on the performance of NPV in the interest of obtaining a minimal false-negative rate given that USS risk stratification functions as a ‘rule-out’ tool that prevents further testing. Additionally, the benefit of NPV is that it takes into account the prevalence of disease.

### Ethics

This study was part of a registered quality improvement project at the institution involved (CEDM Centre of Endocrinology, Diabetes & Metabolism, Limassol) and conformed to the Declaration of Helsinki. Data were pseudo-anonymised prior to statistical analysis. The study received an ethics approval from the Cyprus National Bioethics Committee (ΕΕΒΚ/ΕΠ/2022.01.194) and all participants provided written informed consent.

### Statistics

The continuous variables were presented as means and standard deviation (s.d.) if normally distributed or median and interquartile range (IQR) if not. Correlations between categorical variables were done with chi-square or Fisher’s exact test, as appropriate. Beyond NPV and accuracy, we calculated positive predictive value (PPV), sensitivity, specificity, likelihood ratios and area under the curve (AUC) of receiver operating characteristic (ROC) curves. The formula used for accuracy was as follows: accuracy = (TP+TN)/(TP+TN+FP+FN). These calculations were performed considering all thyroid nodules that were included in our final study cohort, not only nodules with available histopathology. Excel Version 2108 (Microsoft, Washington, USA), IBM SPSS v27 (IBM Corp., NY, USA) and MedCalc Version 20.114 (MedCalc Software Ltd. Ostend, Belgium) were used for statistical analysis.

## Results

**Figure 1:**
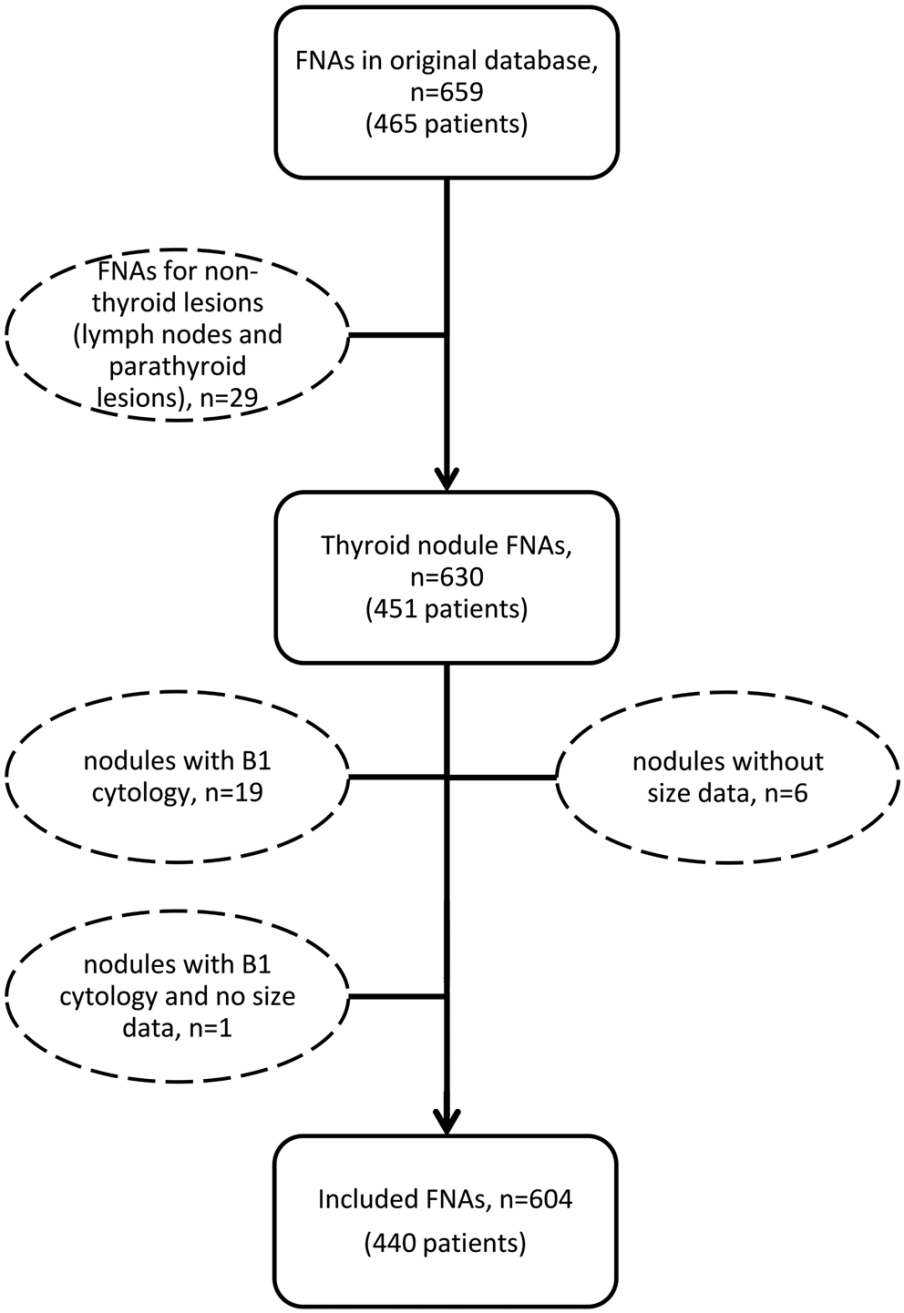
Diagram illustrating the selection of nodules from our original database that were included in the final cohort. FNA, fine-needle aspiration; B1, Bethesda class 1.

**Figure 2:**
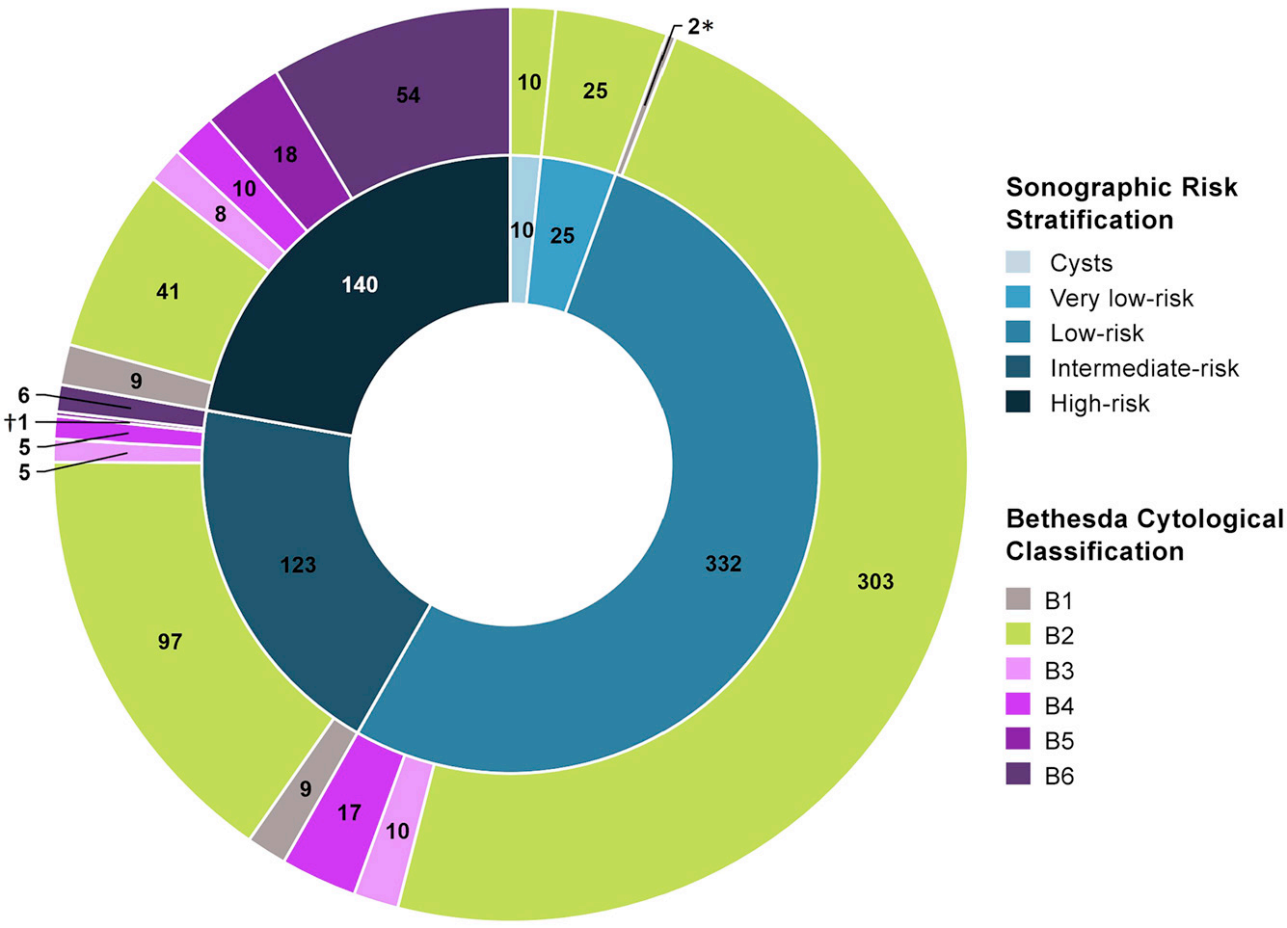
Graph illustrating the correlation between the ultrasound risk stratification and the corresponding cytological grading of the 630 thyroid nodules originally included in the study. ^*^Two low-risk nodules were classified as B1 on cytology. ^†^One intermediate-risk nodule was classified as B5 on cytology.

Our original database included 659 FNA procedures in 465 patients (Fig. 1). After the exclusion of aspirations performed for non-thyroid lesions (lymph nodes or parathyroid lesions), a total of 630 consecutive thyroid nodule FNAs in 451 patients (age 52.0 ± 15.1 years; 77.6% females) were included. Strong sonographic-cytological correlation was observed (*P*-value <0.0001) (Fig. 2). For the nodules with available histopathology, the risk of malignancy was 0, 16.7, 4.8, 36.1 and 75.8% in relation to cystic, very low, low, intermediate and high-risk USS appearances, respectively; the reason for the high percentage in very-low risk nodules is because only six of these nodules underwent surgery. According to the cytological grading of the 630 nodules, the prevalence of each Bethesda class in our population was 3.2% for B1, 75.6% for B2, 3.7% for B3, 5.1% for B4, 3.0% for B5 and 9.5% for B6 and the risk of malignancy was 33.3, 15.7, 22.2, 21.7, 94.7 and 98.0%, respectively. The total rate of malignancy in our study was 14.1% of nodules (89/630), higher than expected in the general population likely owing to the portion of pre-selected patients referred for lymph node mapping. Nodules with non-diagnostic cytology (B1) accounted for only 3.2% of our total examinations (20/630 nodules). Seven nodules (1.1%) lacked adequate documentation of size data, one of which was concomitantly classified as B1. Therefore, 26 nodules (4.1%) were excluded from the final analysis of our results. Our final study cohort consisted of 604 thyroid nodules in 440 patients (age 51.9 ± 15.2 years; 80.0% females). The histopathology reports of 198/604 nodules were available (1 cystic nodule, 6 very low-risk nodules, 62 low-risk nodules, 33 intermediate-risk nodules and 96 high-risk nodules as per the sonographic RSS of the ATA Guidelines). These histologies involved 17 B3 cytologies (previously sonographically stratified as 6 low-risk, 5 intermediate-risk and 6 high-risk nodules) and 23 B4 cytologies (previously sonographically stratified as 13 low-risk, 3 intermediate-risk and 7 high-risk nodules). When the correlation of cytology and histology is considered, the false-positive rate was at 76.5% for B3, 78.3% for B4, 5.3% for B5, 8.0% for B6 and 33.0% for B3–6. Histological examination identified three cases (in two patients) of medullary thyroid carcinoma (MTC), one case of follicular thyroid carcinoma (FTC), one case of invasive encapsulated follicular variant papillary thyroid carcinoma (EFVPTC), one case of oncocytic papillary microcarcinoma while the remainder were classic papillary thyroid carcinomas (PTC). An additional three cases (in two patients) of non-invasive follicular thyroid neoplasm with papillary-like nuclear features (NIFTP) were identified; nevertheless, this is no longer considered a carcinoma.

**Figure 3:**
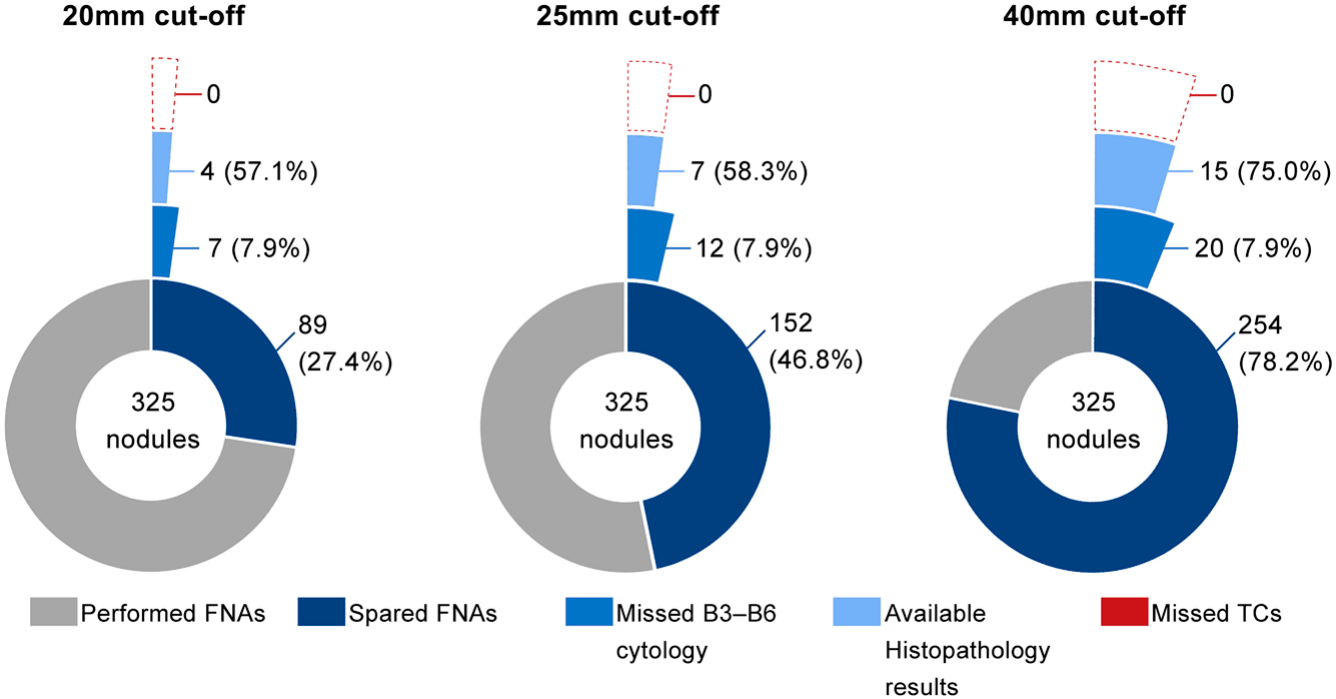
Charts illustrating the proportion of FNAs that would be spared by increasing the ATA size cut-off (15 mm) to each of the higher cut-offs depicted, for sonographically stratified low-risk thyroid nodules. Moreover, the proportion of missed Bethesda classification 3–6 (B3–B6) cytologies among the spared FNAs is shown, along with the number of confirmed thyroid carcinomas (TCs) on biopsy (relative to the number of available histopathology results).

The low, intermediate and high sonographic risk categories were each addressed individually in order to compare the effect of higher size cut-offs on the selection of nodules for FNA (Table 2). In low-risk nodules, NPV remained essentially unchanged with excellent performance around 91% and the accuracy markedly improved from 21.2 to 44.3, 60.6 and 87.1% when the cut-off was increased from 15 to 20, 25 and 40 mm, respectively. The proportion of FNAs that would be spared if the ATA cut-off (15 mm) was modified to the higher cut-offs of 20, 25 (ACR-TIRADS cut-off) and 40 mm would be at 27.4, 46.8 and 78.2%, respectively (Fig. 3). With any of these three higher cut-offs, about 8% of ‘non-benign’ nodules (B3–B6) would have been missed among the spared FNAs. Furthermore, we assessed how many of these missed B3–B6 nodules were histologically determined as carcinomas. Based on our results, by increasing the size cut-offs, no carcinomas would have been missed in the low-risk category. It should be noted that this latter number is relative to the number of histopathology results available since some patients either did not undergo surgery or did not provide their reports.

**Table 2:** Diagnostic performance of ATA size cut-offs against higher cut-offs for low, intermediate and high-risk thyroid nodules.

Sonographic classification^a^	Cut-off (mm)	Sensitivity (95% CI)	Specificity (95% CI)	PPV (95% CI)	NPV (95% CI)	LR+ (95% CI)	LR− (95% CI)	Accuracy (95% CI)	Spared FNAs	Missed B3–B6 cytology	Missed TCs^b^
Low risk (*n* = 325)	15	80.8% (60.7–93.5)	16.1% (12.1–20.7)	7.7% (6.5–9.2)	90.6% (80.7–95.7)	0.96 (0.79–1.17)	1.20 (0.52–2.75)	21.2% (16.9–26.1)			
	20	53.9% (33.4–73.4)	43.5% (37.8–49.3)	7.7% (5.4–10.7)	91.6% (87.5–94.4)	0.95 (0.66–1.38)	1.06 (0.69–1.64)	44.3% (38.8–49.9)	89 (27.4%)	7	0/4
	25	34.6% (17.2–55.7)	62.9% (57.1–68.4)	7.5% (4.5–12.3)	91.7% (89.2–93.7)	0.93 (0.54–1.61)	1.04 (0.78–1.39)	60.6% (55.1–66.0)	152 (46.8%)	12	0/7
	40	3.9% (0.1–19.6)	94.3% (91.1–96.7)	5.6% (0.8–29.8)	91.9% (91.2–92.5)	0.68 (0.09–4.88)	1.02 (0.94–1.11)	87.1% (82.9–90.5)	254 (78.2%)	20	0/15
Intermediate risk (*n* = 113)	10	64.7% (38.3–85.8)	19.8% (12.4–29.2)	12.5% (9.0–17.1)	76.0% (59.7–87.1)	0.81 (0.56–1.16)	1.78 (0.83–3.81)	26.6% (18.7–35.7)			
	15	41.2% (18.4–67.1)	71.9% (61.8–80.6)	20.6% (11.9–33.2)	87.3% (82.0–91.3)	1.46 (0.76–2.81)	0.82 (0.54–1.24)	67.3% (57.8–75.8)	54 (47.8%)	4	0/4
	20	5.9% (0.2–28.7)	89.6% (81.7–94.9)	9.1% (1.4–42.3)	84.3% (82.4–86.0)	0.56 (0.08–4.13)	1.05 (0.92–1.20)	77.0% (68.1–84.4)	77 (68.1%)	10	2/8
High risk (*n* = 131)	10	53.3% (42.5–63.9)	29.3% (16.1–45.5)	62.3% (55.7–68.6)	22.2% (14.5–32.6)	0.75 (0.57–0.99)	1.59 (0.94–2.69)	45.8% (37.1–54.7)			
	15	26.7% (17.9–37.0)	82.9% (67.9–92.9)	77.4% (61.7–88.0)	34.0% (33.0–38.3)	1.56 (0.73–3.33)	0.88 (0.73–1.07)	44.3% (35.6–53.2)	46 (35.1%)	24	15/22
	20	11.1% (5.5–19.5)	87.8% (73.8–95.9)	66.7% (42.2–84.6)	31.0% (28.2–34.0)	0.91 (0.33–2.50)	1.01 (0.88–1.16)	35.1% (27.0–43.9)	62 (47.3%)	38	23/33

The size cut-offs are compared against the cytological result of B2 (‘benign’) or B3–B6 (‘non-benign’).

^a^As per ATA Guidelines; ^b^Relative to number of available histopathology results.

ATA, American Thyroid Association; B3–B6, Bethesda classes 3–6; FNA, fine-needle aspiration; LR−, negative likelihood ratio; LR+, positive likelihood ratio; NPV, negative predictive value; PPV, positive predictive value; TCs, thyroid carcinomas.

**Figure 4:**
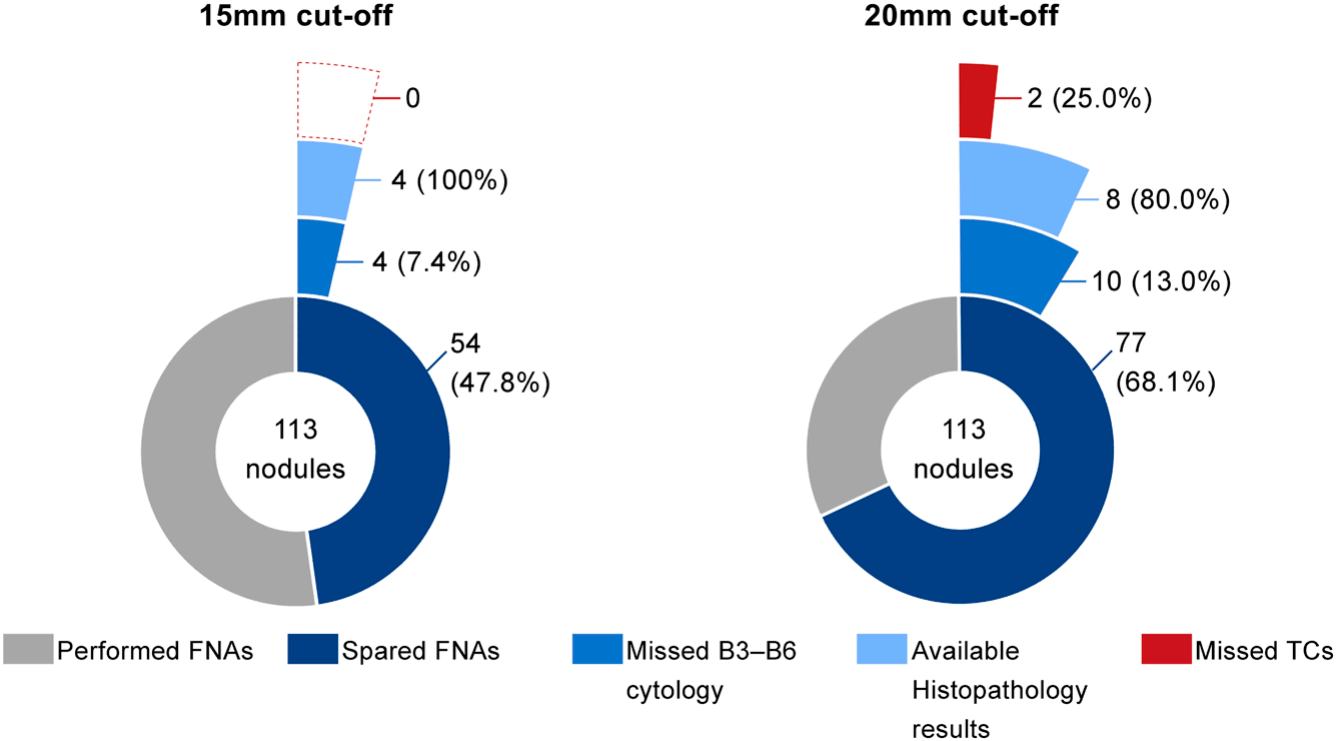
Charts illustrating the proportion of FNAs that would be spared by increasing the ATA size cut-off (10 mm) to each of the higher cut-offs depicted, for sonographically stratified intermediate-risk thyroid nodules. Moreover, the proportion of missed Bethesda classification 3–6 (B3–B6) cytologies among the spared FNAs is shown, along with the number of confirmed thyroid carcinomas (TCs) on biopsy (relative to the number of available histopathology results).

For intermediate-risk nodules, a moderate improvement in NPV is noted from 76.0% at 10 mm to 87.3% at 15 mm and 84.3% at 20 mm, in parallel with quite a considerable increase in accuracy from 26.2 to 67.3 and 77.0%, respectively. Similar to low-risk nodules, raising the threshold by 5 mm and then by 10 mm, a substantial amount of FNAs could be spared (47.8 and 68.1%, respectively), at the expense of a limited proportion of missed B3–B6 cytologies (7.4 and 13.0%, respectively) and carcinomas as demonstrated in Fig. 4. A 10 mm increase in the current size cut-off would have resulted in two missed carcinomas. These two lesions were assigned stage I on diagnosis according to the 8th edition of the AJCC/TNM system (a T1bNxMxR0 FTC and a T1bN0M0R0 PTC).

**Figure 5:**
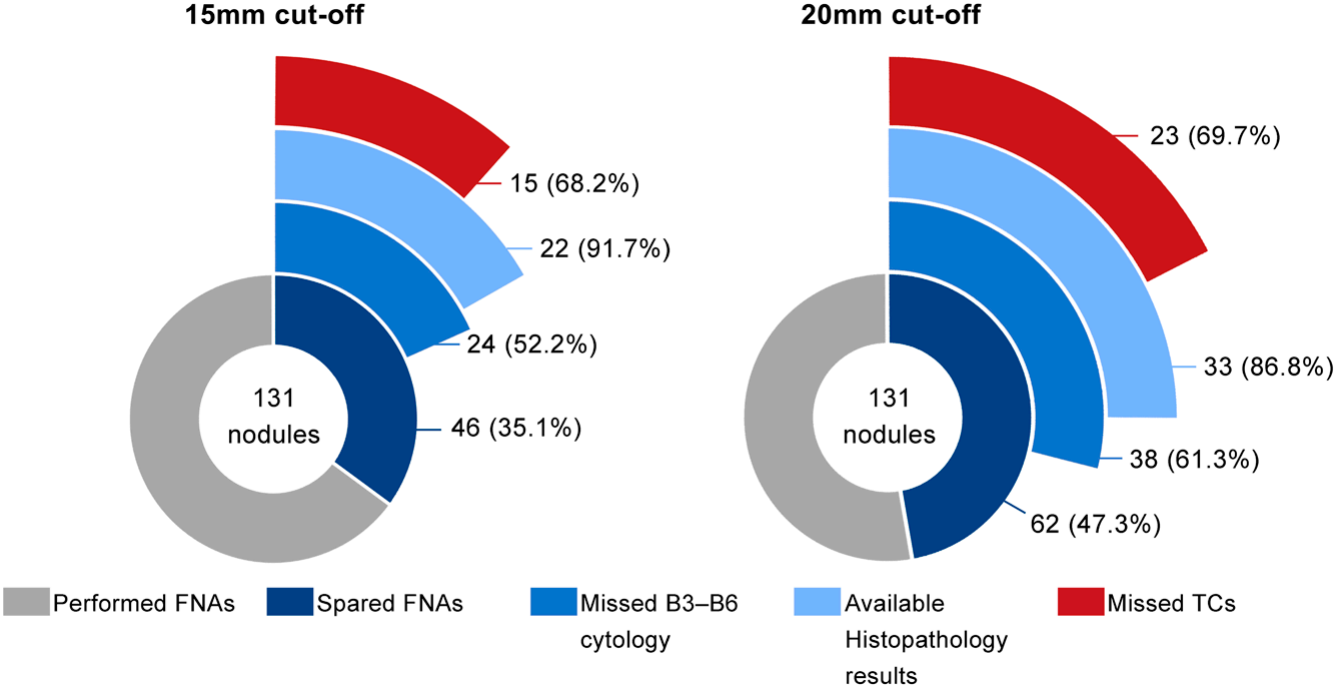
Charts illustrating the proportion of FNAs that would be spared by increasing the ATA size cut-off (10 mm) to each of the higher cut-offs depicted, for sonographically stratified high-risk thyroid nodules. Moreover, the proportion of missed Bethesda classification 3–6 (B3–B6) cytologies among the spared FNAs is shown, along with the number of confirmed thyroid carcinomas (TCs) on biopsy (relative to the number of available histopathology results).

In high-risk nodules, NPV and accuracy perform poorly regardless of cut-off, where the former reaches 22.2% at 10 mm, 34.0% at 15 mm and 31.0% at 20mm, while the accuracy successively declines from 45.8 to 44.3 and 35.1%, respectively. Moreover, a larger proportion of missed B3–B6 cytologies and carcinomas among the spared FNAs was also observed (Fig. 5). Non-benign cytologies were detected in over 50% of the nodules that would not have undergone FNA in case of higher cut-offs. Fifteen and 23 carcinomas would have remained undetected if the cut-off was placed at 15 and 20 mm, respectively.

The AUC of the ROC curves we obtained in all sonographic risk categories and size thresholds we assessed indicate that size does not outperform a ‘random classifier’ with AUC of approximately 0.5.

In the* post hoc* analysis excluding the patients that were referred for pre-operative lymph node mapping, our findings were overall unchanged (Supplementary Table 2).

## Discussion

In low-risk nodules, NPV remains excellent at around 91% and accuracy reaches 87% using higher cut-offs. Using a higher cut-off of 20 or 25 mm, 27 and 47% FNA would have been spared, respectively, without missing any carcinomas. In intermediate-risk nodules, NPV surpasses 80% and accuracy increases by at least 40% with increasing thresholds, while the significant proportion of spared FNAs (48 and 68%) involves no or few (two) missed carcinomas, respectively. Conversely, in high-risk nodules, the increased number of missed ‘non-benign’ cytologies and, indeed, carcinomas could not support any increase of the current 10 mm cut-off.

Our results suggest that by increasing the ATA size cut-off in sonographically low- and intermediate-risk nodules, the high NPV is retained or improved, and accuracy is considerably increased. NPV is unaffected in low-risk nodules as the proportion between the true negative rate (TNR) and the sum of the false negative rate (FNR) and TNR is unchanged at higher thresholds. In intermediate-risk nodules, the increase in the TNR is higher than the increase in the FNR thus resulting in overall increased NPV. The accuracy is improved in both risk categories as the TNR increases substantially with higher cut-offs. Raising the size threshold would result in additional spared FNAs since fewer nodules would meet the increased size cut-offs required for FNA. Considering that the majority of nodules in the low- and intermediate-risk categories were classified as B2 on cytology (92 and 85%, respectively), the spared FNAs mostly concern these ‘benign’ nodules. Therefore, the corresponding increase in the TNR is driving the increase in both NPV and accuracy. Notably, we observed a substantial reduction in the number of FNAs that would have been performed at the expense of a limited proportion of missed B3–B6 cytologies and carcinomas. On the other hand, a higher size cut-off in high-risk nodules results in low NPV and accuracy. Despite the substantial amount of spared FNAs, an unacceptably higher proportion of ‘non-benign’ cytologies and carcinomas would have been missed.

Size is recognized as a poor discriminating factor between benign and malignant thyroid nodules; rather this differentiation ought to be based on other sonographic features i.e. hypoechogenicity, irregular margins, taller-than-wide shape, microcalcifications, capsule infiltration and associated suspicious lymphadenopathy. Consequently, based on our findings, we can argue that the size cut-off should be decided on broad clinical parameters for example, based on the TNM grading system size cut-offs. The higher cut-offs of 20 and 40 mm we assessed may have greater clinical significance, as they correspond to higher tumour grades in case of malignancy (T2 and T3), which could alter the clinical management.

Grani *et al.* showed that among five international classification systems, ACR-TIRADS offers the safest and most significant reduction in FNAs (16). Consistent with our results, a retrospective study by Huh *et al.* which included over 1300 nodules showed an improved diagnostic performance of modified guidelines that simply altered the size cut-offs with the adoption of the ACR-TIRADS thresholds (19). Nonetheless, the ATA Guidelines (10) are widely used as they are user-friendly and less time-consuming presumably because they employ a pattern recognition system rather than a point-scale system. Our results would suggest that the ATA size cut-offs could be safely raised to match those of the EU-TIRADS Guidelines, or even the ACR-TIRADS Guidelines. The low-risk category would benefit from a 5 to 10 mm increase in threshold (as per EU-TIRADS and ACR-TIRADS thresholds, respectively) in safely reducing the number of guideline-indicated FNAs. Likewise, in the intermediate-risk category, both the EU-TIRADS and ACR-TIRADS propose a 5 mm higher size cut-off compared to ATA. According to our evaluation, this threshold offers improved diagnostic performance and no missed carcinomas. Finally, for high-risk nodules, the unanimous cut-off of 10 mm is supported by our data, which demonstrate a higher prevalence of suspicious lesions/malignancy that could be missed if this threshold was raised.

A recent survey administered to physicians of five professional societies addressing the clinical management of thyroid nodules demonstrated the disparity that exists regarding the adoption of ultrasound RSSs in clinical practice (20). This survey was conducted as efforts are currently being made to design one, universal RSS. Aside from the variety of RSSs, some respondents also indicated the concomitant use of more than one system or none at all. The same authors supported that a single, simple, internationally adopted system would prove beneficial to both physicians and patients (20). An accompanying expert commentary highlighted the fact that discordant information between the various RSSs leads to confusion surrounding the management of thyroid nodules especially in primary care physicians and patients (21). Hence, increasing the ATA size cut-offs in line with those proposed by the EU-TIRADS or the ACR-TIRADS would not only improve their diagnostic performance but also reduce discrepancies between available recommendations.

### Strengths and limitations

The strengths of our study include the real-world study design and the prospective data accumulation involving a large number of consecutive thyroid nodules. Furthermore, unlike many previous studies, all cytological classes were included in the analysis of our results (apart from B1). Cytologically benign nodules underwent close follow-up, at least annually, to monitor for any new findings and FNA was performed later if the ATA criteria were fulfilled. Therefore, this reduced the likelihood of a significant amount of false negative ‘benign’ (B2) cytologies.

Nevertheless, our study has some limitations. The reference standard category we employed is not error-free, recognizing the possibility of false results on cytology. In an ‘ideal study’, we would have obtained histopathology diagnoses from all lesions; however, this would not abide by current ethical standards. Moreover, for patients who underwent surgery, the correlation between cytology and histology was excellent (*P*-value <0.0001). Our study has some selection bias given that certain patients were already identified as candidates for thyroid surgery and thus referred to us as a tertiary referral centre for pre-operative lymph node mapping. The* post hoc* analysis did not however give any indications of selection bias arising from these patients referred for pre-operative neck USS assessment. Likewise, we introduced some selection bias because of the exclusion of nodules with non-diagnostic cytology (B1), albeit we strived to keep our exclusion criteria to a minimum. Notwithstanding, the risk of malignancy on histology from our results was comparable to the estimated risk of malignancy reported in the literature (10). An element of attrition bias was also present, consequent to a small number of patients who were lost to follow-up. Finally, in a minority of nodules, the sonographic appearances did not clearly fit a specific ATA category. This was addressed by assigning them on a ‘best-fit’ principle and consensus between all sonographers.

## Conclusion

Based on our results, increasing the size cut-offs of the ATA Guidelines improves the diagnostic performance of the guidelines and results in fewer FNAs being indicated, without significantly increasing the number of missed carcinomas. Our results may reassure doctors and patients alike that low-risk nodules measuring less than 20–25 mm and intermediate-risk nodules measuring less than 15 mm can be managed conservatively, without resorting to FNA biopsy.

## Supplementary Material

Supplementary Table 1. Indications for fine-needle aspiration (FNA) performed in the 91 nodules measuring less than 10mm.

Supplementary Table 2. Post-hoc analysis excluding nodules from patients referred for pre-operative lymph node mapping.

## Declaration of interest

The authors declare that there is no conflict of interest that could be perceived as prejudicing the impartiality of the research reported.

## Funding

This work did not receive any specific grant from any funding agency in the public, commercial or not-for-profit sector.

## Author contribution statement

A K conceived the study. A K, G Z and M P recruited the patients and recorded the clinical information. S I wrote the first draft. S I and A K performed the statistical analysis. All authors reviewed, contributed and approved the final manuscript.
