# Supplementary material for: Real-world application of ATA Guidelines in over 600 aspirated thyroid nodules: is it time to change the size cut-offs for FNA?
Source: Eur Thyroid J. 2022 Oct 10;11(6):e220163. doi: 10.1530/ETJ-22-0163 (PMC9641794; doi:10.1530/ETJ-22-0163)
Supplement: Supplementary Table 1. Indications for fine-needle aspiration (FNA) performed in the 91 nodules measuring less than 10mm. [file supplementary_table_1.pdf]

Supplementary Table 1. Indications for fine-needle aspiration (FNA) performed in the 91 nodules measuring less than 10mm.

Reason for FNA in nodule <10mm	No. of nodules (n=91)
Suspicion of thyroid capsule infiltration on USS	34
FNA performed in another nodule fulfilling size criteria in contralateral thyroid lobe and done to facilitate selection of surgical approach (total vs hemi-thyroidectomy)	21
Suspicious lymphadenopathy	11
Family history of thyroid carcinoma	7
Patient preference	7
Suspicion of capsule infiltration and indication for FNA in nodule in contralateral lobe	6
Sudden appearance and growth of nodule	2
Patient referred for parathyroidectomy and FNA of associated thyroid nodules was advised by MDT	2
Patient was renal transplant donor and FNA of an associated thyroid nodule was advised prior to nephrectomy	1

USS, ultrasound scan. MDT, multidisciplinary team.
