# Supplementary material for: Real-world application of ATA Guidelines in over 600 aspirated thyroid nodules: is it time to change the size cut-offs for FNA?
Source: Eur Thyroid J. 2022 Oct 10;11(6):e220163. doi: 10.1530/ETJ-22-0163 (PMC9641794; doi:10.1530/ETJ-22-0163)
Supplement: Supplementary Table 2. Post-hoc analysis excluding nodules from patients referred for pre-operative lymph node mapping. [file supplementary_table_2.pdf]

Supplementary Table 2. Post-hoc analysis excluding nodules from patients referred for pre-operative lymph node mapping.

		Cut-off	NPV (95% CI)	Accuracy (95% CI)	Spared FNAs	Missed B3–B6 cytology	Missed TCs†
**Sonographic classification as per ATA guidelines**	**Low-risk** (n=318)	**15mm**	97.9% (87.2-99.7)	21.4% (17.0-26.3)			
		**20mm**	94.2% (90.1-96.6)	45.0% (39.4-50.6)	89 (28.0%)	7	0/4
		**25mm**	93.5% (90.9-95.3)	61.3% (55.7-66.7)	151 (47.5%)	12	0/7
		**40mm**	93.0% (92.4-93.6)	88.1% (84.0-91.4)	252 (79.2%)	20	0/15
	**Intermediate-risk** (n=105)	**10mm**	94.4% (71.4-99.1)	25.7% (17.7-35.2)			
		**15mm**	93.1% (87.4-96.3)	69.5% (59.8-78.1)	54 (51.4%)	4	0/4
		**20mm**	89.4% (87.3-91.1)	81.0% (72.1-88.0)	76 (72.4%)	9	2/7
	**High-risk** (n=87)	**10mm**	47.8% (31.3-64.9)	54.0% (43.0-64.8)			
		**15mm**	52.5% (45.7-59.2)	58.6% (47.6-69.1)	38 (43.7%)	17	9/15
		**20mm**	46.0% (41.7-50.3)	48.3% (37.4-59.3)	51 (58.6%)	28	16/24

The size cut-offs are compared against the cytological result of B2 ('benign') or B3–B6 ('non-benign'). †Relative to number of available histopathology results. ATA, American Thyroid Association. NPV, negative predictive value. FNA, fine-needle aspiration. B3–B6, Bethesda classes 3–6. TCs, thyroid carcinomas.
